# The influence of sleep apnea syndrome and intermittent hypoxia in carotid adventitial vasa vasorum

**DOI:** 10.1371/journal.pone.0211742

**Published:** 2019-02-05

**Authors:** Carolina López-Cano, Ferran Rius, Enric Sánchez, Anna Michela Gaeta, Àngels Betriu, Elvira Fernández, Andree Yeramian, Marta Hernández, Marta Bueno, Liliana Gutiérrez-Carrasquilla, Mireia Dalmases, Albert Lecube

**Affiliations:** 1 Endocrinology and Nutrition Department, University Hospital Arnau de Vilanova, Obesity, Diabetes and Metabolism (ODIM) research group, IRBLleida, University of Lleida, Lleida, Catalonia, Spain; 2 Respiratory Department, University Hospital Arnau de Vilanova-Santa María, Translational Research in Respiratory Medicine, IRBLleida, University of Lleida, Lleida, Catalonia, Spain; 3 Unit for the Detection and Treatment of Atherothrombotic Diseases (UDETMA V&R), University Hospital Arnau de Vilanova, Vascular and Renal Translational Research Group, IRBLleida, University of Lleida, Lleida, Catalonia, Spain; 4 Centro de Investigación Biomédica en Red de Enfermedades Respiratorias (CIBERES), Instituto de Salud Carlos III (ISCIII), Madrid, Spain; 5 Centro de Investigación en Red en Diabetes y Enfermedades Metabólicas Asociadas (CIBERDEM), Instituto de Salud Carlos III (ISCIII), Madrid, Spain; International University of Health and Welfare, School of Medicine, JAPAN

## Abstract

Subjects with sleep apnea-hypopnea syndrome (SAHS) show an increased carotid intima-media thickness. However, no data exist about earlier markers of atheromatous disease, such as the proliferation and expansion of the adventitial vasa vasorum (VV) to the avascular intima in this setting. Our aim was to assess carotid VV density and its relationship with sleep parameters in a cohort of obese patients without prior vascular events. A total of 55 subjects evaluated for bariatric surgery were prospectively recruited. A non-attended respiratory polygraphy was performed. The apnea-hypopnea index (AHI) and the cumulative percentage of time spent with oxygen saturation below 90% (CT90) were assessed. Serum concentrations of soluble intercellular adhesion molecule 1, P-selectin, lipocalin-2 and soluble vascular cell adhesion molecule 1 (sVCAM-1) were measured. Contrast-enhanced carotid ultrasound was used to assess the VV density. Patients with SAHS (80%) showed a higher adventitial VV density (0.801±0.125 vs. 0.697±0.082, p = 0.005) and higher levels of sVCAM-1 (745.2±137.8 vs. 643.3±122.7 ng/ml, p = 0.035) than subjects with an AHI lower than 10 events/hour. In addition, a positive association exist between mean VV density and AHI (r = 0.445, p = 0.001) and CT90 (r = 0.399, p = 0.005). Finally, in the multiple linear regression analysis, female sex, fasting plasma glucose and AHI (but not CT90) were the only variables independently associated with the mean adventitial VV density (R^2^ = 0.327). In conclusion, a high VV density is present in obese subjects with SAHS, and chronic intermittent hypoxia is pointed as an independent risk factor for the development of this early step of atheromatous disease.

## Introduction

There is considerable evidence that obesity is a causal factor for sleep-disordered breathing (SDB) with more than 50% of patients having a body mass index (BMI) greater than 30 Kg/m^2^ [1, 2]. Sleep apnea-hypopnea syndrome (SAHS), the most common type of SDB, has been well established as an independent risk factor for some components of the metabolic syndrome, such as hypertension and glucose abnormalities, along with other cardiovascular risk factors [3, 4]. In this regard, early signs of atherosclerosis, such as an increase in carotid intima-media thickness (cIMT), have been reported in patients with SAHS without other cardiovascular diseases [5]. In fact, observational studies have shown that patients with SAHS have an increased risk of death, especially from stroke and myocardial infarction [6, 7]. However, the correction of nocturnal chronic intermittent hypoxia (CIH) using continuous positive airway pressure (CPAP) has not been associated with a decreased risk of cardiovascular outcomes or death for patients with SAHS [8, 9]. These data increase the need for studies that describe better all mechanisms by which intermittent hypoxia damages the endothelial wall.

Atherosclerosis, a chronic inflammatory disorder of the wall of large and medium arteries, initiates several decades before reaching clinical significance [10]. Although the cIMT represents an early surrogate marker for atherosclerosis, an increasing amount of evidence supports the hypothesis that the atheromatosis process begins with the hyperplasia and pathological extension of the adventitial vasa vasorum (VV) to the avascular intima [11, 12]. The VV are a network of microvessels physiologically located in the third and most external wall coat of the medium and large arteries. Their main function is to supply the required nutrients and oxygen to the vessel cells [12, 13]. However, its proliferation and expansion from the adventitial layer is the earliest defensive response to endothelial injury by deleterious stimuli, such as hypoxia, inflammation, and hyperglycemia [11, 12, 14–17]. In fact, the lack of correlation between cIMT and adventitial VV density in clinical studies support the hypothesis that both measures evaluate different moments in the development of atheromatous disease [17, 18]. In the last decade, the contrast-enhanced ultrasound (CEU) has emerged as a useful and non-invasive technique for direct visualization of the VV in the carotid arteries. Consequently, CEU represent a novel approach to detect the development of premature stages of atherosclerosis [18–20] which has not been previously used to explore the relationship between SDB and carotid VV density.

To shed light in the deleterious impact of nocturnal intermittent hypoxia in carotid wall, we have assessed the carotid adventitial VV density and its relationship with the sleep study in a cohort of obese patients without any prior vascular event evaluated for bariatric surgery.

## Material and methods

### Description of the study population

A total of 55 morbidly obese patients of Caucasian origin without previous episodes of vascular disease were prospectively recruited for the study between January 2016 and July 2017 at the outpatient Obesity Unit. All the candidates were contacted during one of the visits in the outpatient clinic or by telephone once the sleep breathing evaluation included in the established protocol for bariatric surgery was available. If the patient was willing to participate, we gave them detailed information about the objectives and procedures of the study.

Using the standard deviation of adventitial VV from a previous study, we determined that the minimum necessary sample size was 35 subjects [21]. Therefore, the study surveyed a total of 69 consecutive individuals who met the eligibility criteria for gastrointestinal surgery established by the guidelines of the National Institutes of Health Consensus Conference [22]. We excluded 12 patients for the following reasons: treatment with CPAP (n = 7), previous medical history of any cardiovascular event (n = 3), a former bariatric procedure (n = 1), and a glomerular filtration rate lower than 60 ml/min/per 1.73 m^2^ (n = 1). Additionally, two patients were excluded for technical problems (fast clearance of the contrast that did not allow a proper assessment of VV density) from the final analysis. No pregnant women were included.

### Ethic statement

The study was approved by the human ethics committee at Arnau de Vilanova University Hospital (CEIC-1275) and was conducted according to the ethical guidelines of the 1964 Helsinki Declaration and its later amendments. Informed written consent was obtained from all participants included in the study.

### Measurement of sleep-disordered breathing

A non-attended respiratory polygraphy was performed at patient’s home with a Somnea polygraph (Compumedics, Abbotsford, Australia) which records nasal airflow (nasal cannula), respiratory effort (chest and abdominal bands), snoring, body position and finger pulse oximetry [23, 24]. The same technician manually checked all sleep studies to avoid variability. Studies with less than 5 hours of correct signal recording were discarded and repeated. An apnea was defined as cessation of airflow for more than 10 seconds. Differentiation between obstructive and central apneas was based according to the respiratory effort channels and the presence or absence of thoracoabdominal movements. Hypopnea was defined as a decrement in nasal cannula tracing of at least 50% with duration of at least 10 seconds, which is associated with a cyclical dip in arterial oxygen saturation of 4% or more [25]. The apnea-hypopnea index (AHI) was defined as the sum of apneas plus hypopneas divided by the recording time in bed. On this basis, SAHS was defined as an AHI ≥ 10 events/hour (e/h), and patients were divided in non-SAHS (AHI < 10 e/h), mild SAHS (AHI between 10 and 20 e/h), moderate SAHS (AHI between 21 and 30 e/h), and severe SAHS (AHI>30 e/h) [26]. The cumulative percentage of time spent with oxygen saturations below 90% (CT90) was also assessed. In addition, the degree of sleepiness was evaluated by using the Epworth Sleepiness Scale, a widely-used questionnaire assessment of the tendency to fall asleep during various daytime situations [27].

### Measurement of the carotid adventitial VV density and ultrasound parameters

CEU examinations were completed using a Siemens Sequoia 512 ultrasound system (equipped with a 15L8W linear array probe) and with ultrasound contrast software (Cadence contrast Pulse Sequencing technology). A phospholipidic shell containing sulphur hexafluoride served as a contrast agent (Sonovue, Bracco Spa, Milan, Italy). Once the contrast was solubilized in 5 ml of saline, a 2.5 ml bolus was injected in the antecubital vein for each carotid artery explored (20-gauge needle to avoid microbubbles rupture). Adventitial VV content in the far adventitial layer was calculated as the average of the ratios of the intensities in the 2 mm above the intima-lumen boundary and the intensities of the 2 mm below the media-adventitia boundary of the common carotid artery 1 cm proximal to the bifurcation. The result (VV signal) was calculated as the average of 10 to 20 ratios calculated for each diastolic frame in which both the lumen intensity and the adventitial intensity was high and stable within a 1-minute video recording [21, 28]. Results are displayed on the right and left sides, and the mean VV signal of both sides is presented. As a ratio, VV signal has no units. All the studies were stored digitally for a posterior analysis by the same blinded investigator. Additionally, all participants underwent a B-mode ultrasound examination of the extra-cranial carotid arteries and the cIMT of the far wall of the common carotid artery was measured following the Mannheim consensus procedures.

### Laboratory assessment

Blood sampling by direct puncture of the antecubital vein was obtained after an overnight fast of 8 hours and just before administration of the contrast agent. Samples were separated by centrifugation (2.000 g at 4°C for 20 min) and analyzed in the clinical laboratory of our hospital using standard methods to obtain biochemical parameters. In addition, aliquots were frozen at -80°C for batched analysis. Serum concentrations of soluble intercellular adhesion molecule 1 (sICAM-1), P-Selectin, lipocalin-2 and soluble vascular cell adhesion molecule 1 (sVCAM-1) were measured in duplicate using the Human Cardiovascular Disease Magnetic Bead Panel 2 from Milliplex Map Kit (Cat. No. HCVD2MAG-67K, Billerica, MA).

Blood pressure was measured with an automated oscillometer (Omron HEM-705CP) as the average of three readings separated by one minute after ten minutes of rest. Normal blood pressure was diagnosed when participants not receiving antihypertensive treatment had a blood pressure below 120/80 mmHg.

### Statistical analysis

Normal distribution of the variables was assessed using the Kolmogorov-Smirnov test. Data were expressed either as the mean ± standard deviation or percentage. Given their skewed distribution, AHI, CT90 and serum triglycerides are shown as median (total range). For parametric tests, all three parameters were logarithmically transformed to achieve a normal distribution. Comparisons between groups were performed using Student *t* tests and ANOVA testing for continuous variables, and the *χ*^2^ test for categorical variables. The relationship between the continuous variables was examined by the Pearson linear correlation test.

A stepwise multiple regression analysis was performed in a backward direction to explore the variables independently related to VV. The independent variables included in the analyses were related with polysomnographic parameters (AHI and CT90), clinically relevant with potential impact in atheromatous disease [age, sex, body mass index (BMI), systolic and diastolic blood pressure, HDL and LDL-cholesterol, and smoking status (current or former smoker vs. non-smoker)], and significantly associated with the mean VV density in the univariate analysis (triglycerides, fasting plasma glucose, and neck and waist circumferences). The significance level at which independent variables were removed from the model was a F-test value <0.05.

All *p* values were based on a two-sided test of statistical significance. Significance was accepted at the level of *p* < 0.05. Statistical analyses were performed using the SPSS statistical package (IBM SPSS, Statistics for Windows, Version 20.0. Armonk, NY, USA).

## Results

The main clinical features and metabolic data of the study population are presented in **Table 1**. A total of 40 patients (72.7%) were diagnosed with some degree of SAHS: 14 (35.0%) with mild, 6 (15.0%) with moderate and 20 (50.0%) with severe SAHS. In addition to a higher daytime sleepiness, subjects with SAHS were older and presented a higher prevalence of type 2 diabetes than patients without SAHS.

**Table 1:** Baseline main clinical, metabolic and sleep-breathing characteristics of participants in the study according to the diagnosis of sleep apnea-hypopnea syndrome.

	All patients	SAHS	Non-SAHS	p
**N**	55	40	15	-
**Women, n (%)**	29 (70.9)	26 (65.0)	13 (86.6)	0.184
**Age (years)**	46.1 ± 10.9	47.8 ± 11.7	41.7 ± 7.0	0.023
**BMI (Kg/m**^**2**^**)**	44.4 ± 6.6	44.8 ± 7.1	43.0 ± 5.3	0.370
**Waist circumference (cm)**	127.0 ± 14.9	128.6 ± 13.7	123.0 ± 16.0	0.211
**Neck circumference (cm)**	40.8 ± 5.2	41.4 ± 5.4	39.0 ± 4.1	0.118
**Current/former smoker (%)**	34 (61.8)	25 (62.5)	9 (60.0)	0.865
**Type 2 diabetes, n (%)**	17 (30.9)	16 (40.0)	1 (6.6)	0.022
**FPG (mmol/l)**	6.3 ± 2.1	6.6 ± 2.5	5.6 ± 0.7	0.033
**Hypertension, n (%)**	23 (41.8)	19 (47.5)	4 (26.6)	0.224
**Systolic BP (mmHg)**	125.9 ± 17.8	126.9 ± 17.9	123.1 ± 18.0	0.481
**Diastolic BP (mmHg)**	77.9 ± 11.9	77.4 ± 12.9	79.1 ± 9.0	0.652
**Triglycerides (mmol/l)**	1.5 (0.1–4.3)	1.6 (0.7–4.3)	1.3 (0.7–2.5)	0.302
**LDL-cholesterol (mmol/l)**	2.7 ± 0.8	2.6 ± 0.8	2.9 ± 0.7	0.261
**HDL-cholesterol**	1.1 ± 0.2	1.1 ± 0.3	1.2 ± 0.1	0.228
**ESS**	4.1 ± 4.7	4.9 ± 5.2	1.5 ± 1.2	0.012
**AHI (events/hour)**	20.0 (1.0–83.0)	31.5 (10.0–83.0)	5.0 (1.0–8.0)	<0.001
**CT90 (%)**	9.0 (0–88.0)	12.0 (0.1–88.0)	1.0 (0–14.0)	<0.001

Data are mean ± SD, median (range) or n (percentage). SAHS: sleep apnea-hypopnea syndrome, BMI: body mass index; FPG: fasting plasma glucose; BP: blood pressure; LDL: low density lipoprotein; ESS: Epworth Sleepiness Scale; AHI: apnea-hypopnea index; CT90: percentage of time spent with oxygen saturations below 90%.

The mean adventitial VV density was significantly higher in patients with SAHS compared to subjects with an AHI <10 e/h (0.801 ± 0.125 *vs*. 0.697 ± 0.082, p = 0.005). This result was also observed on the right and left sides (p = 0.038 and p = 0.027, respectively) (**Table 2**). A progressive increase in the mean adventitial VV density was observed from participants without SAHS to those with mild (0.757 ± 0.097), moderate (0.865 ± 0.199) and severe (0.811 ± 0.111) SAHS (p = 0.008, respectively). However, no differences were appreciated between subjects with and without SAHS when the mean cIMT was evaluated (0.743 ± 0.127 *vs*. 0.683 ± 0.133, p = 0.613). Among serum markers of endothelial damage, patients with SAHS showed significantly higher levels of sVCAM-1 than subjects with an AHI lower than 10 e/h (742.8 ± 132.7 vs. 636.8 ± 120.5, p = 0.012). The serum concentration of VCAM-1 also exhibited a progressive increase throughout SAHS severity: 636.8 ± 120.5 (non-SAHS), 699.1 ± 124.5 (mild), 799.9 ± 79.1 (moderate) and 761.9 ± 145.9 (severe) ng/ml (p ANOVA = 0.029). Subjects without SAHS showed serum VCAM-1 concentrations significantly lower than patients with moderate (p = 0.013) and severe (p = 0.016) SAHS.

**Table 2:** Carotid adventitial VV density, cIMT and serum biomarkers of endothelial damage of the individuals included in the study according to the presence of sleep apnea-hypopnea syndrome.

	SAHS	Non-SAHS	Mean difference (95% CI)	p
**N**	40	15	-	-
**Mean VV**	0.801 ± 0.125	0.697 ± 0.082	0.103 (0.033–0.173)	0.005
**Right side VV**	0.829 ± 0.164	0.729 ± 0.115	0.099 (0.005–0.193)	0.038
**Left side VV**	0.750 ± 0.124	0.665 ± 0.113	0.085 (0.010–0.160)	0.027
**Mean cIMT (mm)**	0.743 ± 0.127	0.683 ± 0.133	0.060 (-0.018–0.139)	0.128
**sICAM-1 (ng/ml)**	100.4 ± 37.8	104.0 ± 42.5	-3.6 (-28.4 to 21.2)	0.771
**P-Selectin (ng/ml)**	123.0 ± 42.5	123.6 ± 34.3	-0.5 (-29.0 to 27.8)	0.967
**Lipocalin-2 (ng/ml)**	176.4 ± 77.0	194.8 ± 66.0	-18.3 (-65.7 to 28.9)	0.438
**sVCAM-1 (ng/ml)**	742.8 ± 132.7	636.8 ± 120.5	105.9 (23.9 to 188.0)	0.012

Data are mean ± SD. SAHS: sleep apnea-hypopnea syndrome. cIMT: carotid intima-media thickness; sICAM1: soluble intercellular adhesion molecule 1; sVCAM-1: soluble vascular cell adhesion molecule 1.

**Fig 1:**
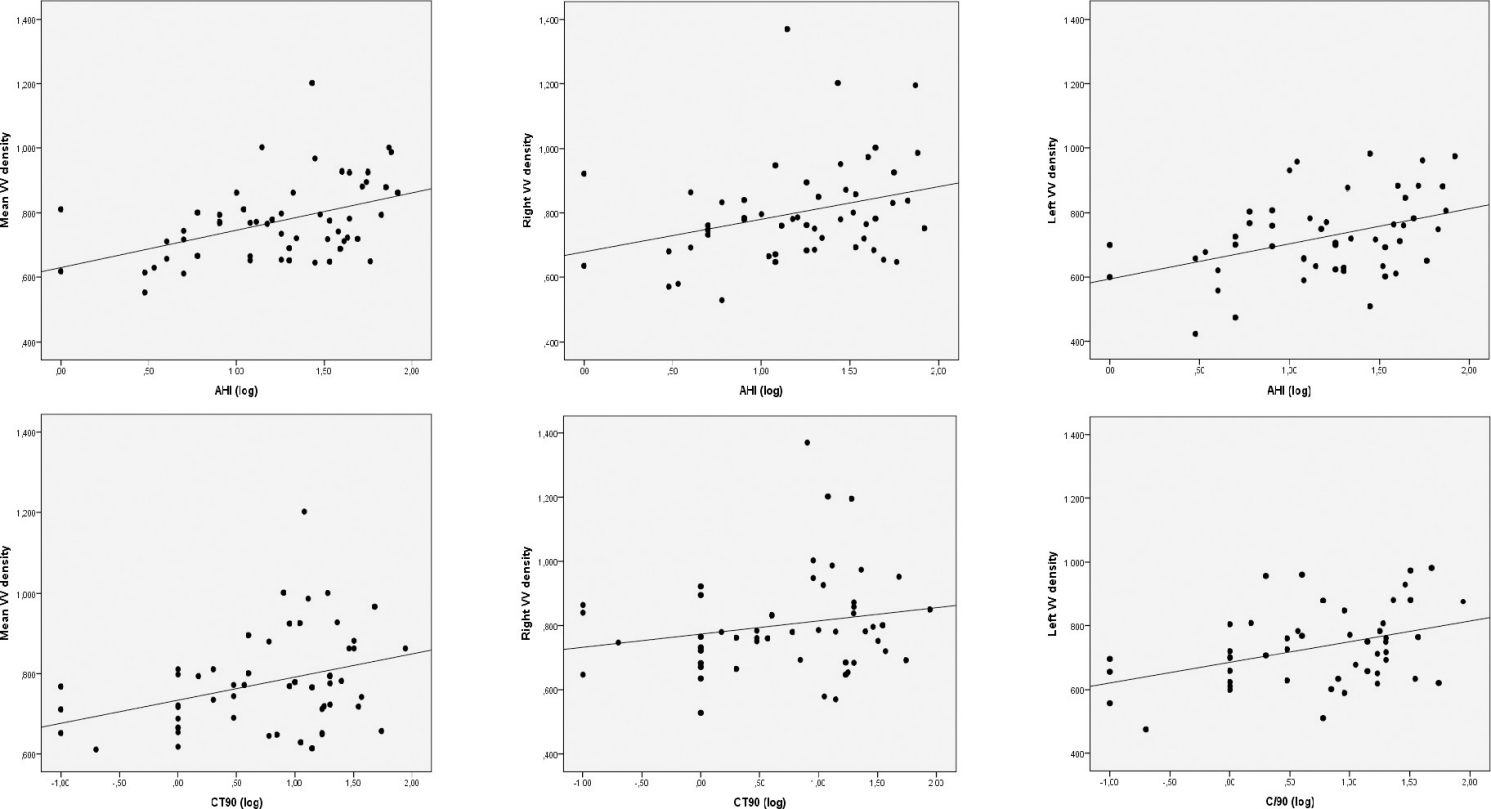
Correlations of the apnea-hypoapnea index (AHI) and the percentage of time spent with oxygen saturations below 90% (CT90) with vasa vasorum (VV) density (mean, right side and left side).

Univariate analysis showed a positive and significant association between the mean adventitial VV density and the AHI (log) (r = 0.445, p = 0.001) and CT90 (log) (r = 0.399, p = 0.005) (**Fig 1**). The associations between carotid VV density and both nocturnal hypoxia parameters were maintained for the left side (AHI: r = 0.408, p = 0.003; CT90: r = 0.469, p = 0.001) but partially disappeared for the right one (AHI: r = 0,302, p = 0.029; CT90: r = 0,222, p = 0.142, respectively). We also found significant correlations between the mean VV density and fasting plasma glucose (r = 0.321, p = 0.017), triglycerides (log) (r = 0.304, p = 0.030), neck (p = 0.003, p = 0.388) and waist circumferences (p = 0.028, r = 0.296), as well as with the serum concentration of sVCAM-1 (r = 0.345, p = 0.014). However, no significant association between the mean adventitial VV density and total cholesterol (r = 0.062, p = 0.668), HDL-cholesterol (r = 0.160, p = 0.268) and LDL-cholesterol (r = 0.110, p = 0.446) was found.

Finally, the multiple linear regression analysis showed that female sex, fasting plasma glucose and AHI (but not CT90, blood pressure, lipid profile, smoking status, age and anthropometric indices) were the only variables independently associated with the mean adventitial VV density (R^2^ = 0.401) (**Table 3).**

**Table 3:** Stepwise multiple linear regression analysis of variables associated with the mean adventitial VV density.

		β	Beta (95% CI)	p
**Mean VV density**	**Sex (female/male)**	0.394	0.104 (0.038 to 0.170)	<0.001
	**FPG (mmol/l)**	0.294	0.015 (0.003–0.028)	0.015
	**AHI (log)**	0.257	0.067 (0.002 to 0.132)	0.043
	**LDL-cholesterol mmol/l)**	0.204	-	0.081
	**Triglycerides (log)**	0.172	-	0.150
	**HDL-cholesterol (log)**	-0.124	-	0.307
	**Age (years)**	-0.132	-	0.311
	**Smoking status** ^**a**^	-0.117	-	0.333
	**BMI (kg/m**^**2**^**)**	0.106	-	0.394
	**CT90 (log)**	0.098	-	0.466
	**Diastolic BP (mmHg)**	0.069	-	0.557
	**Waist circumference (cm)**	0.045	-	0.731
	**Systolic BP (mmHg)**	-0.028	-	0.817
	**Neck circumference (cm)**	-0.005	-	0.977
R^2^ = 0.401	Constant	-	0.467 (0.346 to 0.589)	<0.001

β: standardized coefficient; Beta: non-standardized coefficient; FPG: fasting plasma glucose; AHI: apnea-hypopnea index; CT90: percentage of time spent with oxygen saturations below 90%; BMI: body mass index; BP: blood pressure

^a^ current/former smoker *vs*. non-smoker. Equation for multiple regression: [Mean VV density = 0.467 + 0.104 x sex (female = 1, male = 0) + 0.015 x FPG (mmol/l) + 0.067 x AHI (log)].

## Discussion

To the best of our knowledge, this is the first study to establish a relationship between repetitive nocturnal upper airway obstructions and adventitial VV density, one of the earlier steps in the development of atheromatous disease. We also provide evidence that the CT90% correlates with VV density in the univariate analysis. However, this late characterization of the severity of nocturnal hypoxemia disappears as an independent variable of the onset of atheromatous disease in the multivariate regression analysis.

SAHS is a highly prevalent disease characterized by cyclical episodes of desaturation-reoxygenation associated with higher carbon dioxide levels and sleep disruption [29]. It is also a key mediator of cardiac and vascular disease and dysfunction and appears as an independent risk factor for all-cause and cardiovascular mortality [6, 7, 30, 31]. Accordingly, large population observations and prospective studies have consistently shown a gradual increase in the prevalence of cardiovascular events as the AHI increases [30–32]. A recent meta-analysis of 17 prospective cohort studies reported that the incidence of cardiovascular disease was significantly increased in moderate-severe SAHS, with a pooled relative risk compared to the reference group of 1.37 (0.95–1.98) for coronary heart disease and 2.02 (1.40–2.90) for stroke [33]. This association persisted after controlling for classic vascular risk factors such as type 2 diabetes, hypertension, smoking habit and dyslipidemia [34]. In addition, data from the *Sleep Heart Health Study* reinforced the critical role of intermittent hypoxia. They described how hypopneas, defined by a threshold of 4% or more decrease in oxygen saturation, were independently associated with self-reported prevalent cardiovascular disease [35].

The presence of coronary artery calcification (CAC), as a surrogate of subclinical coronary artery disease, was evaluated on 202 consecutive subjects who underwent an overnight-sleep study [36]. CAC was found in 67% of patients with an AHI ≥5 e/h in comparison with the 31% in non-SAHS patients (p = 0.001), with a progressive increase in median CAC score with SAHS severity [36]. In the same vein as this result, we found that AHI was an independent predictor of the mean VV density in multiple regression analysis.

The underlying causes by which nocturnal intermittent hypoxia initiates and worsens arterial neovascularization have not been fully elucidated. A few pathophysiological mechanisms have been suggested to participate in the endothelial injury, such as insulin resistance and glucose intolerance, disruptions in the pathways of inflammation and in the function of macrophages, lipid metabolism defects and sympathetic activation [34, 37, 38]. Although each one may explain a part of the whole picture, we believe many of them act in chorus in subjects with SAHS. In this regard, severe nocturnal oxyhemoglobin desaturations have been associated with high fasting levels of very low-density lipoprotein [39]. In addition, CIH also activates the production of reactive oxygen species (ROS) that generate systemic inflammation and endothelial dysfunction through the induction of cytokines and cell adhesion molecules [40–42]. Our results demonstrated an increase in serum concentration of sVCAM-1 among subjects with SAHS, and its measurement had a positive and significant correlation with the carotid VV density. Actually, in superoxide dismutase deficient mice, an animal model of increased oxidative stress, sVCAM-1 plays an important role in its increased susceptibility to severe ocular neovascularization [43]. In other tissues, such as the endothelium of the large and medium arteries, ROS also activates the hypoxia-inducible factor 1a (HIF-1a), which mediates angiogenesis induction of vascular endothelial growth factor and nitric oxide synthase genes [14, 44]. The role of VCAM-1 in the development of atheromatous disease in patients with SAHS is also supported by data showing how intermittent hypoxia produces increased expression in coronary artery endothelial cells [45]. Similarly, the lectin-like oxidized low-density lipoprotein receptor-1 (LOX-1), a single transmembrane receptor mainly expressed on endothelial cells that mediates the uptake of oxidized LDL and also plays an important role in ischemia-induced angiogenesis, upregulates the expression of VCAM [46].

Experimental evidence supports that the expansion of the adventitial vessels to the intima layer plays a central role in the initiation and progression of the atherosclerotic process. The appearance of new blood vessels in the intima would facilitate the extravasation and accumulation in the outer intimal of blood-born oxidized low-density lipoprotein particles that will facilitate plaque initiation and formation [47, 48]. Therefore, the evaluation of VV appears to be a more accurate tool to reflect earlier stages of atherosclerosis, even before the development of an increased cIMT. Our data support this concept, as carotid adventitial VV density progressively increases together with AHI severity in subjects without previous vascular disease. Finally, the exposure to both nocturnal hypoxia patterns may exert differential modulation of the hemodynamic and hemorheology responses. In morbidly obese subjects AHI appears to be a better predictor of VV density than sleep measurements related with the overall time spent in severe hypoxemia and classical vascular risk factors such as age, lipid profile, tobacco, obesity and blood pressure [49, 50].

Although AHI appears as an independent predictor of the mean VV density in the multiple linear regression analysis, by itself it has a limited impact on the overall variance of this variable. We believe it is important to highlight this fact in order to achieve a better interpretation of our results. Our study has also identified other factors that influence the mean VV density, such as female sex and fasting plasma glucose. Undoubtedly, all these variables act jointly and probably synergistically in the initial development of atheromatous disease. The importance of our study lies in the addition of intermittent hypoxia to the most classic cardiovascular risk factors.

With the carotid adventitial VV imaging we focus our attention on the onset of atheromatous disease, when the pathological changes are potentially reversible. Our group has recently documented a significant 12.0% decrease in mean carotid adventitial VV density after the marked reduction in body weight and the improvement of the main metabolic comorbidities achieved after bariatric surgery [18]. In addition, in a novel animal model, the early structural cardiovascular remodeling induced by intermittent hypoxia was normalized after resumption of normoxic breathing [51]. Whether changes observed after CPAP treatment, as the improvement in postprandial triglycerides and total cholesterol levels, the favorable effect on antioxidant capacity and the enhancement in cardiovascular risk biomarkers may also have a direct impact in carotid VV in patients with SAHS still needs to be elucidated [52–54].

Our study has some limitations. First, we have not performed full polysomnographies but a home non-attended respiratory polygraphies. Therefore, we have no data about other key sleep features with a potential impact in cardiovascular disease such as sleep latency, microarousals, changes in stage 2 sleep and rapid eye movement sleep. In addition, the AHI was calculated from time in bed, not from sleeping time, and could underestimate the prevalence of SAHS. Second, our results are unlikely to change current practices in SAHS because the CEU examination is not routinely available in clinical practice and is rather expensive and time consuming. However, the opportunity of recognizing the arteriosclerotic process in its early stages may be used to implement preventive strategies to reduce the risk of future cardiovascular events in selected vulnerable subjects with SAHS. Third, the high percentage of women included in our study (70.9% of our sample) makes difficult to generalize our results to the entire population. However, this percentage is similar to 74.2% of women among obese subjects who underwent bariatric surgery in public Spanish hospitals between years 2000 and 2014 [55].

In conclusion, we observe a high VV density in obese subjects with SAHS, and we find that CIH is an independent risk factor for the development of this precocious remodeling of the arterial wall in the setting of atheromatous disease. We believe that the measurement of this cardiovascular risk marker, preceding intimal thickening and atherosclerotic plaque formation, is a good tool to achieve a better assessment of subjects with SAHS and increased vascular risk. However, specific studies in patients with SAHS are needed to determine whether a precocious detection and intervention (for example, with CPAP treatment) on this form of subclinical atheromatous disease could prevent or slow down the development of cardiovascular events in this population.

## Supporting information

S1 File Excel database including the main data from participants in the study. (XLSX)
